# Supplementary material for: Tunable localization of light using nested invisible metasurface cavities
Source: Nanophotonics. 2023 Feb 20;12(6):1083–9. doi: 10.1515/nanoph-2022-0549 (PMC11501781; doi:10.1515/nanoph-2022-0549)
Supplement: Supplementary file 1 — Supplementary Material Details [file j_nanoph-2022-0549_suppl.pdf]

Supplementary Material: Tunable Localization of Light Using Nested Invisible Metasurface Cavities

S1. EFFECT OF LOSSES IN AN INVISIBLE CAVITY

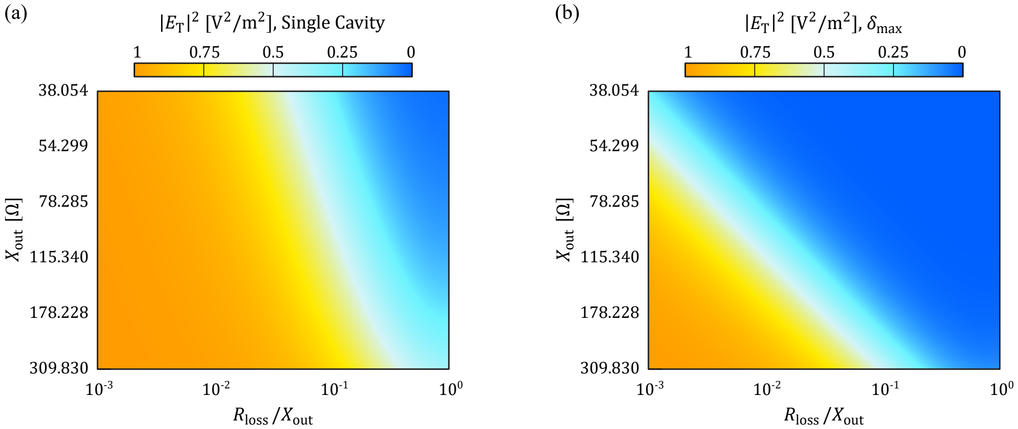

Fig. S1. (a) When inherent losses are taken into account, performance of a single invisible cavity degrades when the resistive part of the sheet impedance becomes comparable to the walls' reactance, making the device opaque. In the case of a nested cavity, the performance depends on the relative location of the inner cavity. (b) The cavity is more sensitive to loss when it is aligned to the electric field maxima of the outer cavity at δ_{max}. In both cases, an incident wave with $E_I = 1$ V/m is assumed.

Ideally, the invisible cavity is conceived as a lossless device, whose inductive and capacitive sheets cancel the phase shifts introduced by each other. If the cavity is based on active and lossy sheets, the power absorbed by one of the sheets should be compensated by the active one. However, real passive metasurfaces have inherent losses (in the chosen materials), which cannot be compensated for cavities ideally designed as lossless. This behaviour can be observed in Fig. S1(a), where a single invisible cavity is made of lossy metasurfaces. For simplicity, we assume that both metasurfaces have the same surface resistance R_{loss}, defined as proportional to the surface reactance $|X_{out}|$. As the cavity becomes more resonant (with stronger localized field), it also becomes less tolerant to the intrinsic metasurface loses, resulting in a device that attenuates the amplitude of the transmitted wave.

This analysis can be also extended for nested configurations. The loss effects in a combination of two lossy invisible cavities, is presented in Fig. S1(b). When the inner cavity is aligned with the electric field maxima at δ_{max}, the device becomes more sensitive to the effect of inherent losses. In either case, but especially for the setup aligned at δ_{max}, the intensity of the transmitted wave decays as the loss resistance becomes comparable to the surface reactance.

S2. INVISIBLE CAVITY RESONATOR UNDER COHERENT ILLUMINATION

An invisible cavity resonator is a combination of two impedance sheets with the relation $Z_{e1} = -Z_{e2}$, separated by a multiple of half-wavelengths at the resonant frequency ($d = n\lambda_0/2$ with n being an integer value) [1]. At the resonance frequency, under plane-wave illumination, the fields along the structure are given by

$$\mathbf{E}_I = E_I e^{-jk_0 z}\mathbf{a}_x, \tag{S1a}$$

$$\mathbf{E}_{F,I} = E_I\left(1 - \frac{\eta_0}{2Z_{e1}}\right)e^{-jk_0 z}\mathbf{a}_x, \tag{S1b}$$

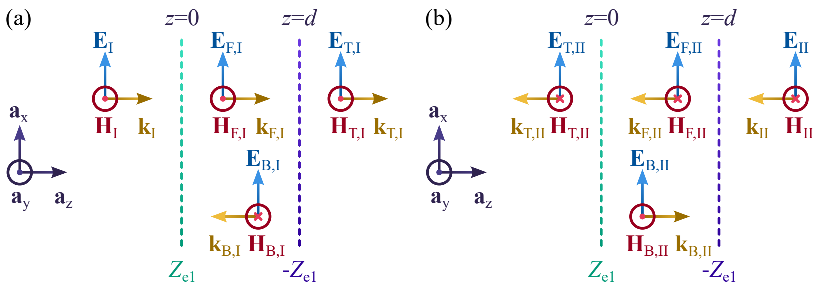

Fig. S2. (a) An invisible cavity can support a standing wave without creating external scattering and without adding any delay into the transmitted wave. (b) The cavity remains invisible regardless from which end it is illuminated.

$$\mathbf{E}_{\mathrm{B,I}} = E_{\mathrm{I}} \left(\frac{\eta_0}{2Z_{\mathrm{e1}}} \right) e^{jk_0 z} \mathbf{a}_{\mathrm{x}}, \tag{S1c}$$

$$\mathbf{E}_{\mathrm{T,I}} = E_{\mathrm{I}} e^{-jk_0 z} \mathbf{a}_{\mathrm{x}}, \tag{S1d}$$

where η_0 is the characteristic impedance of the medium around the impedance sheets (assumed to be free space in this work), and k_0 is the wavenumber at the resonant frequency. The standing wave inside the cavity can be characterized in terms of field maxima and minima, which read

$$E_{\mathrm{max,I}} = A_{\mathrm{max,I}} \left| E_{\mathrm{I}} \right| = \left| \frac{E_{\mathrm{I}}}{2Z_{\mathrm{e1}}} \right| \left(\left| 2Z_{\mathrm{e1}} - \eta_0 \right| + \eta_0 \right), \tag{S2a}$$

$$E_{\mathrm{min,I}} = A_{\mathrm{min,I}} \left| E_{\mathrm{I}} \right| = \left| \frac{E_{\mathrm{I}}}{2Z_{\mathrm{e1}}} \right| \left(\left| 2Z_{\mathrm{e1}} - \eta_0 \right| - \eta_0 \right). \tag{S2b}$$

Illumination from the other end of the cavity, as shown in Fig. S2(b), will produce a different standing wave inside, whose fields are given by

$$\mathbf{E}_{\mathrm{II}} = E_{\mathrm{II}} e^{jk_0 z} \mathbf{a}_{\mathrm{x}}, \tag{S3a}$$

$$\mathbf{E}_{\mathrm{F,II}} = E_{\mathrm{II}} \left(1 + \frac{\eta_0}{2Z_{\mathrm{e1}}} \right) e^{jk_0 z} \mathbf{a}_{\mathrm{x}}, \tag{S3b}$$

$$\mathbf{E}_{\mathrm{B,II}} = E_{\mathrm{II}} \left(-\frac{\eta_0}{2Z_{\mathrm{e1}}} \right) e^{-jk_0 z} \mathbf{a}_{\mathrm{x}}, \tag{S3c}$$

$$\mathbf{E}_{\mathrm{T,II}} = E_{\mathrm{II}} e^{jk_0 z} \mathbf{a}_{\mathrm{x}}. \tag{S3d}$$

In the scenario of Fig. S3(b), where the invisible cavity resonator is illuminated from both sides, the fields inside the cavity are the linear combination of the standing waves produced by each individual incident wave:

$$\begin{aligned} \mathbf{E}_{\mathrm{F,comb}} &= \mathbf{E}_{\mathrm{F,I}} + \mathbf{E}_{\mathrm{B,II}} \\ &= \left[E_{\mathrm{I}} \left(1 - \frac{\eta_0}{2Z_{\mathrm{e1}}} \right) - E_{\mathrm{II}} \left(\frac{\eta_0}{2Z_{\mathrm{e1}}} \right) \right] e^{-jk_0 z} \mathbf{a}_{\mathrm{x}}, \end{aligned} \tag{S4a}$$

$$\begin{aligned} \mathbf{E}_{\mathrm{B,comb}} &= \mathbf{E}_{\mathrm{B,I}} + \mathbf{E}_{\mathrm{F,II}} \\ &= \left[E_{\mathrm{I}} \left(\frac{\eta_0}{2Z_{\mathrm{e1}}} \right) + E_{\mathrm{II}} \left(1 + \frac{\eta_0}{2Z_{\mathrm{e1}}} \right) \right] e^{jk_0 z} \mathbf{a}_{\mathrm{x}}. \end{aligned} \tag{S4b}$$

Additional insights can be obtained if the second incident wave is defined in terms of the first one as

$$E_{\mathrm{II}} = \rho e^{j\phi} E_{\mathrm{I}}, \tag{S5}$$

where ρ is the amplitude ratio and ϕ is the phase difference. In that case, the amplitude of the combined forward and backward waves can be expressed as

$$E_{\mathrm{F,comb}} = \frac{E_{\mathrm{I}}}{2Z_{\mathrm{e1}}} \left[2Z_{\mathrm{e1}} - \eta_0 \left(1 + \rho e^{j\phi} \right) \right], \tag{S6a}$$

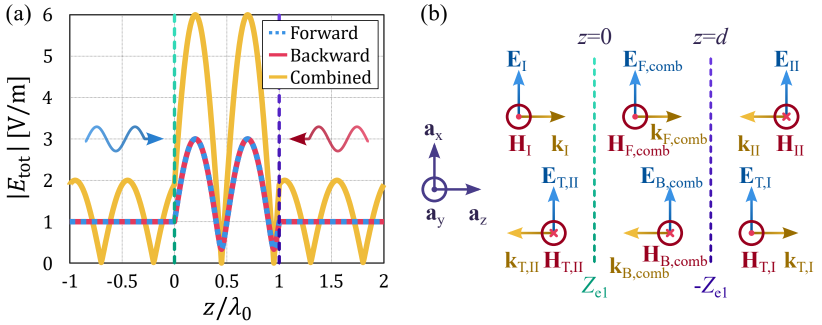

Fig. S3. (a) A lossless invisible cavity can support the same standing wave inside it, regardless from which direction it is illuminated. With the proper phase difference of the coherent sources, the standing wave can be combined constructively. (b) As long as both surface impedances have a linear response, the fields created under coherent illumination are the combination of the fields excited individually by each source.

$$E_{\mathrm{B,comb}} = \frac{E_{\mathrm{I}}}{2Z_{\mathrm{e1}}}\left[\eta_0 + \rho e^{j\phi}\left(2Z_{\mathrm{e1}} + \eta_0\right)\right]. \tag{S6b}$$

In terms of field localization, it becomes relevant to determine the right combination of incident waves that maximizes the amplitude of the standing wave. In that sense, the phase difference ϕ is the parameter of choice, as the amplitude ratio ρ only determines which incident wave is dominant in terms of power contribution. In fact, the optimal phase difference ϕ_{opt} does not depend on the amplitude ratio when the resonator is bounded by lossless walls ($Z_{\mathrm{e1}} = jX_{\mathrm{e1}}$). In that particular case, the standing wave's amplitude is maximized when ϕ_{opt} satisfies the relation

$$e^{j\phi_{\mathrm{opt}}} = \frac{\eta_0 - 2jX_{\mathrm{e1}}}{|\eta_0 - 2jX_{\mathrm{e1}}|}, \tag{S7}$$

producing the standing wave of Fig. S2(d) with the field maximum value

$$\begin{aligned} E_{\mathrm{max,comb}} &= \left|\frac{E_{\mathrm{I}}}{2X_{\mathrm{e1}}}\right| \left(\eta_0 + |\eta_0 - 2jX_{\mathrm{e1}}|\right)(1+\rho) \\ &= A_{\mathrm{max,I}}\,|E_{\mathrm{I}}|\,(1+\rho)\,. \end{aligned} \tag{S8}$$

S3. COHERENT ILLUMINATION OF NESTING CAVITIES

Illuminating an invisible cavity resonator from its both ends can be done easily by placing it inside a larger resonant cavity, which in this case of study can be another invisible cavity as shown in Fig. S4(a). In this setup, the inner resonator is fed with the standing wave contained inside the outer resonator. Since the outer standing wave illuminates the inner resonator with a different phase, the standing wave of the innermost resonator is affected by its relative position, as shown in Fig. S4(b). Using the resonator wall located at $z = \delta$ as the reference coordinates in z-axis, the amplitude of the coherent sources read

$$E_{\mathrm{I,in}} = E_{\mathrm{F,out}} e^{-jk_0\delta} = E_{\mathrm{I}}\left(1 - \frac{\eta_0}{2Z_{\mathrm{out}}}\right) e^{-jk_0\delta}, \tag{S9a}$$

$$E_{\mathrm{II,in}} = E_{\mathrm{B,out}} e^{jk_0\delta} = E_{\mathrm{I}}\left(\frac{\eta_0}{2Z_{\mathrm{out}}}\right) e^{jk_0\delta}. \tag{S9b}$$

Therefore, the relation between sources of Eq. (S5) can be reduced into the form

$$\rho e^{j\phi} = \frac{E_{\mathrm{II,in}}}{E_{\mathrm{I,in}}} = \frac{\eta_0}{2Z_{\mathrm{out}} - \eta_0} e^{2jk_0\delta}, \tag{S10}$$

whose phase component reads

$$e^{j\phi} = \frac{|2Z_{\mathrm{out}} - \eta_0|}{2Z_{\mathrm{out}} - \eta_0} e^{2jk_0\delta}. \tag{S11}$$

Assuming a lossless inner cavity ($Z_{\mathrm{in}} = jX_{\mathrm{in}}$), the strongest field localization is achieved at δ_{max},

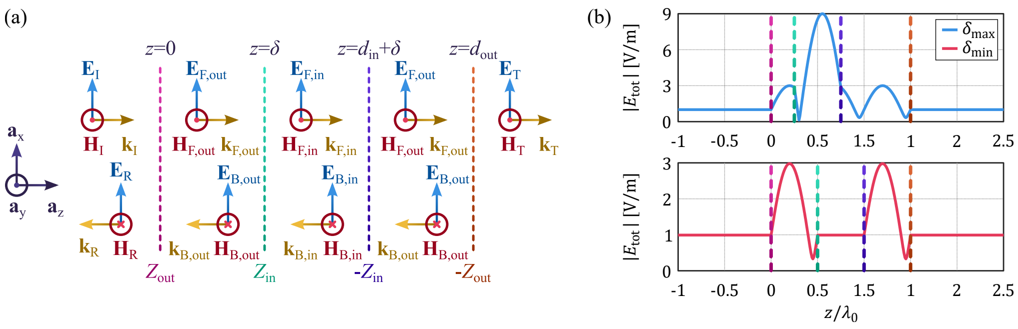

Fig. S4. (a) A nested invisible cavity is the combination of two individual invisible cavities, one inside the other, located at a distance δ from the wall of the outer cavity. (b) The standing wave of the inner cavity can be controlled using its relative location δ, achieving strong field localization or even just a travelling wave.

resulting of matching the optimal-phase coherent illumination of the inner cavity from Eq. (S7) with the phase shift in the outer standing wave components of Eq. (S11):

$$e^{2jk_0\delta_{\text{max}}} = -\frac{\eta_0 - 2jX_{\text{in}}}{|\eta_0 - 2jX_{\text{in}}|}\frac{\eta_0 - 2Z_{\text{out}}}{|\eta_0 - 2Z_{\text{out}}|}, \tag{S12}$$

with the peak field maximum

$$\begin{aligned} E_{\text{max,peak}} &= |E_{\text{I}}| \frac{(\eta_0 + |\eta_0 - 2jX_{\text{in}}|)(\eta_0 + |\eta_0 - 2Z_{\text{out}}|)}{|4X_{\text{in}}Z_{\text{out}}|} \\ &= |E_{\text{I}}| A_{\text{max,in}} A_{\text{max,out}}. \end{aligned} \tag{S13}$$

S4. NUMERICAL CALCULATION OF THE QUALITY FACTOR FOR A NESTED CAVITY

When the nested cavity of Fig. S4(a) is illuminated with a source at an arbitrary frequency f, invisibility of the combined structure is not guaranteed, except for the resonant frequency f_0. Under this assumption, the transmitted wave should be calculated enforcing the boundary conditions at each cavity sheet. Each metasurface is modelled as a sheet maintaining electric surface current with sheet impedance Z_n, which follows the relations

$$\mathbf{a}_z \times (\mathbf{E}_+ - \mathbf{E}_-) = 0, \tag{S14a}$$

$$\mathbf{a}_z \times (\mathbf{H}_+ - \mathbf{H}_-) = \mathbf{J}_n, \tag{S14b}$$

$$\mathbf{J}_\text{e} = \frac{\mathbf{E}_+ + \mathbf{E}_-}{2Z_n}, \tag{S14c}$$

where $\mathbf{E}_+$ ($\mathbf{H}_+$) is the net tangential electric (magnetic) field after the n-th metasurface (with respect to the direction of $\mathbf{a}_z$, orthogonal to the sheet), $\mathbf{E}_-$ ($\mathbf{H}_-$) is the net tangential electric (magnetic) field before the interface, $\mathbf{J}_n$ is the induced surface current density on the sheet, proportional to the averaged tangential electric fields on the interface [2]. The resulting transmitted wave for the nested cavity of Fig. S4(a) can be written as

$$E_\text{T} = \frac{16\tilde{Z}_{\text{in}}^2\tilde{Z}_{\text{out}}^2 e^{2jk(\delta + d_{\text{in}} + d_{\text{out}})}}{\Delta} E_\text{I}, \tag{S15a}$$

$$\begin{aligned} \Delta =& e^{2jk\delta}\left[e^{4jkd_{\text{in}}} + \left(4\tilde{Z}_{\text{in}}^2 - 1\right)\left(4\tilde{Z}_{\text{out}}^2 - 1\right)e^{2jk(d_{\text{in}}+d_{\text{out}})} + \left(4\tilde{Z}_{\text{in}}^2 - 1\right)e^{2jkd_{\text{in}}} + \left(4\tilde{Z}_{\text{out}}^2 - 1\right)e^{2jkd_{\text{out}}}\right] \\ &+ \left(1 - e^{2jkd_{\text{in}}}\right)\left[(2\tilde{Z}_{\text{in}}+1)(2\tilde{Z}_{\text{out}}+1)e^{2jk(2\delta+d_{\text{in}})} + (2\tilde{Z}_{\text{in}}-1)(2\tilde{Z}_{\text{out}}-1)e^{2jkd_{\text{out}}}\right], \end{aligned} \tag{S15b}$$

where $\tilde{Z}$ is the normalized value of the surface impedance with respect to the medium characteristic impedance η_0 ($Z = \tilde{Z}\eta_0$) and k is the wavenumber of the incident wave. By assuming

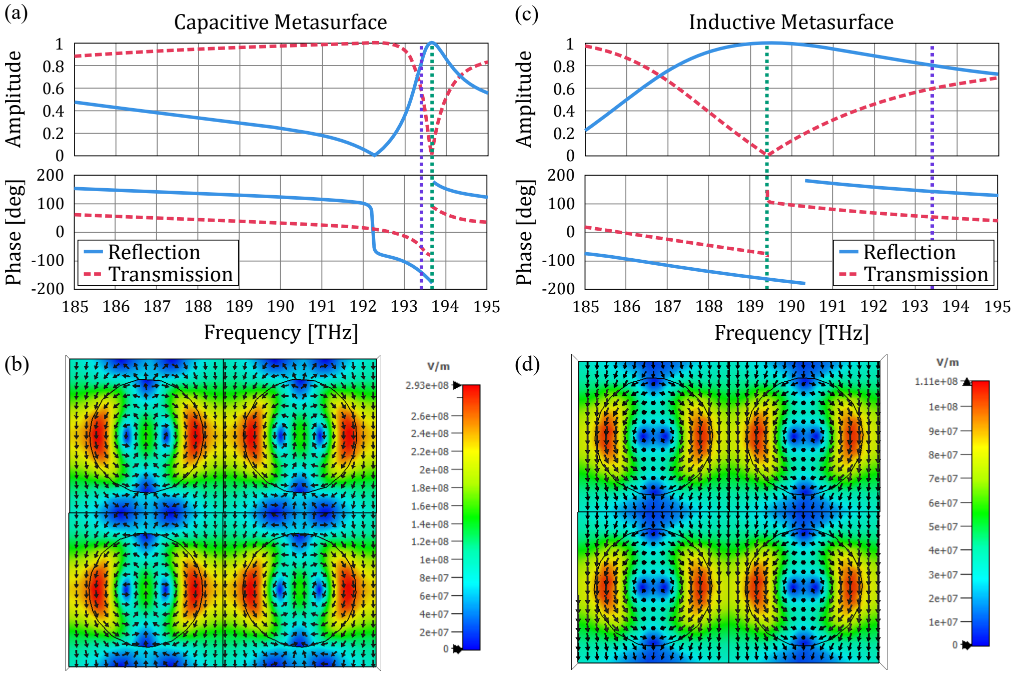

Fig. S5. (a) Frequency response of the capacitive metasurface, exhibiting PEC behavior at 193.65 THz (green dotted line). The design frequency of a metasurface cavity, 193.41 THz, is shown by the violet dotted line. (b) The electric field distribution at the cross-section parallel to the capacitive metasurface (crossing nanodisks in halves) at the resonance frequency of 193.65 THz. Four unit cells are shown. The electric field resembles that of an array of electric dipoles (the dipoles are centered between neighboring nanodisks), confirming that the metasurface operates at the electric-type resonance. (c) Frequency response of the inductive metasurface, exhibiting PEC-type scattering at 189.43 THz (green dotted line). (d) Same as (b) but for inductive metasurface at its resonance frequency of 189.43 THz.

that the nested cavity is a combination of alternating lossless inductive and capacitive layers ($Z_{\text{out}} = -Z_{\text{in}} = jX_{\text{out}}$), the transmitted wave can be reduced to the form

$$E_{\text{T}} = \frac{16\tilde{X}_{\text{out}}^4 e^{2jk(\delta+d_{\text{in}}+d_{\text{out}})}}{\Delta} E_{\text{I}}, \tag{S16a}$$

$$\begin{aligned}\Delta =& e^{2jk\delta}\left[e^{4jkd_{\text{in}}} - \left(1+4\tilde{X}_{\text{out}}^2\right)\left(e^{2jkd_{\text{in}}} + e^{2jkd_{\text{out}}}\right) + \left(1+4\tilde{X}_{\text{out}}^2\right)^2 e^{2jk(d_{\text{in}}+d_{\text{out}})}\right] \\ &+ \left(1-e^{2jkd_{\text{in}}}\right)\left(1+4\tilde{X}_{\text{out}}^2\right)\left(e^{2jk(2\delta+d_{\text{in}})} + e^{2jkd_{\text{out}}}\right).\end{aligned} \tag{S16b}$$

S5. EFFECTIVE SCATTERING FROM SINGLE NANODISK LAYERS

In order to design an invisible cavity, it is required to have a pair of metasurfaces with matched scattering. It was found in previous works, like Ref. [1], that the field localization grows as the scattering of the metasurfaces tends asymptotically to the one of PEC (zero transmission with reflection coefficient equal to -1). Based on this information, the inductive and capacitive metasurfaces can be designed from the same primitive metasurface, which has PEC-behavior at the design frequency [$f_0 = 193.41$ THz in this case – violet dotted line in Fig. S5(a),(c)]. Then, by changing the metasurface parameters (the radius and height of the nanodisk), the PEC resonance is displaced to a higher frequency for the capacitive metasurface, as shown in Fig. S5(a). On the other hand, and as presented in Fig. S5(c), the inductive metasurface is obtained by displacing the resonance to a lower frequency. Therefore, at the operational frequency (where the nested cavity becomes invisible, $f = 193.695$ THz), both metasurfaces would have scattering with the same amplitude but with opposite phase (being positive for the inductive metasurface and negative for the capacitive one). The induced fields around the nanodisks correspond to electric-dipole

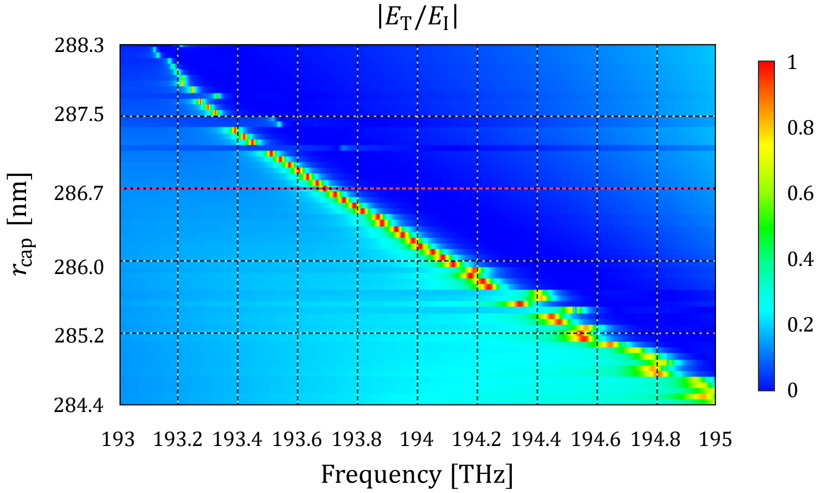

Fig. S6. Variations of the nanodisk radius would produce shifts in the operational frequency. In this scenario, the nested cavity is tested using capacitive nanodisks with different radii. The original radius is shown by the magenta dashed line.

resonance, as shown in Fig. S5(b) for the capacitive metasurface and in Fig. S5(d) for the inductive one.

S6. EFFECT OF NANODISK VARIATION IN NESTED CAVITY PERFORMANCE

Imperfections and fabrication constraints would affect the performance of a single invisible cavity and, by extension, the resulting nesting of two or more cavities. In order to characterize these effects, the nested cavity was tested for different nanodisk radii. More accurately, the radius of the capacitive nanodisk was assumed to take different values between 284.4–288.3 nm, with a span around 4 nm which is expected for current state-of-the-art fabrication [3]. The capacitive radius r_{cap} (radius of nanodisks in capacitive metasurface) was chosen for the characterization as this metasurface is more sensitive to small changes, as its resonant frequency is the closest to the operational frequency. The CST results of Fig. S6, obtained by placing the inner cavity at $\delta_{\max} = 1.68\lambda_0$, show that the operational frequency (where the cavity is transparent) shifts as a function of r_{cap}. However, this radii variation can be considered negligible as the operational frequency only shifts in a span of 2 THz – about 1% of the operational frequency. Notably, the transmission amplitude remains close to 1 within the mentioned deviation range of r_{cap}.

REFERENCES

[1] F. S. Cuesta, V. S. Asadchy, A. D. Sayanskiy, V. A. Lenets, M. S. Mirmoosa, X. Ma, S. B. Glybovski, and S. A. Tretyakov, "Nonscattering metasurface-bound cavities for field localization, enhancement, and suppression," *IEEE Transactions on Antennas and Propagation*, vol. 68, no. 3, pp. 1689–1703, 2020.

[2] S. Tretyakov, *Analytical Modeling in Applied Electromagnetics*. Boston: Artech House, 2003.

[3] N. Levanon, S. R. K. C. Indukuri, C. Frydendahl, J. Bar-David, Z. Han, N. Mazurski, and U. Levy, "Angular transmission response of in-plane symmetry-breaking quasi-bic all-dielectric metasurfaces," *ACS Photonics*, vol. 9, no. 11, pp. 3642–3648, 2022.
